# Which questionnaires can be used to elicit patients’ preferences regarding patient-provider consultations? Results of a scoping review

**DOI:** 10.1186/s12913-025-12567-2

**Published:** 2025-04-04

**Authors:** Alina Zoe Bambas, Diana Wahidie, Yüce Yilmaz-Aslan, Patrick Brzoska, Claudia Kiessling

**Affiliations:** 1 Education of Personal and Interpersonal Competencies in Health Care, Faculty of Health, Witten/Herdecke University, Alfred-Herrhausen-Str. 50, Witten, 58455 Germany; 2 Department Health Services Research, Faculty of Health, Witten/Herdecke University, Witten, Germany

**Keywords:** Patient preferences, Patient-provider consultation, Patient-centered care, Patient-provider communication, Shared decision making, Providing information, Scoping review, Questionnaires

## Abstract

**Objective:**

Active patient involvement and attention to patient preferences in patient-provider consultations are increasingly recognized as essential to improve patient satisfaction and outcomes. Aim of the review was to provide an overview of questionnaires that measure patient preferences regarding communication, information provision and involvement in decision-making in patient-provider consultations.

**Methods:**

Inclusion criteria were studies that provided primary data, were published in German or English, and included adult patients. The systematic search was conducted in PubMed and PsycInfo. Data extraction and summary focused on information about the development process, topic and structure, and reliability of instruments.

**Results:**

Of 6,667 abstracts screened, 34 articles were included, describing 37 different instruments, often originating from an Anglo-American context. Twelve articles reported patient involvement in the development process. Majority of questionnaires measures aspects of information and/or decision-making preferences. Fewer instruments focus on patient-centeredness or communication preferences. Length of questionnaires varied from one to 80 items. Only 15 studies reported reliability indices.

**Conclusion:**

Due to the heterogeneous description, a more consistent reporting of data would be desirable for future publications as well as more participatory research.

**Practice implications:**

Although there is a wide range of questionnaires available, more research is needed to determine the extent to which they can be used in everyday clinical practice to elicit preferences from individual patients with a wide range of conditions and cultural backgrounds.

**Supplementary Information:**

The online version contains supplementary material available at 10.1186/s12913-025-12567-2.

## Introduction

Societal expectations about the provider-patient relationship and consultation have changed considerably over the last few decades [1]. A systematic review by Chewning et al. [2] found that a majority of patients prefer to share decisions with doctors, indicating a tendency towards a more active role in decision-making. They also found a trend over time, with increasing tendency of a shared decision-making process in studies from 2000 onwards. Therefore, healthcare providers have been encouraged to involve patients more actively as partners in the decision-making process [3–5] and become more patient-centered in the way they organize patient contacts [6, 7]. Patients’ preferences for participation in treatment processes are given greater consideration [8–10]. This is particularly important for patients with chronic conditions compared to patients with acute conditions who have a good chance of recovery due to, for example, prolonged and repeated contact with doctors, the specific role of coping and self-management, and long-term psychosocial burden.

An overarching model for these concerns is a patient-centered medicine [11–13], which embraces—according to Mead & Bower [14]—the consideration of a biopsychosocial perspective [15], the concept of patient and doctor as ‘persons’, the sharing of power and responsibility, and the notion of the doctor-patient-relationship as a therapeutic alliance.

Patient-centeredness becomes particularly evident in the patient-provider consultation, e.g. in the way the health care provider initiates a session, gathers information as part of the medical history, shares information, advices on changes in lifestyle and involves a patient in decision making processes. In this context and against the background of an increasing awareness of patient rights, demands from patients and researchers arose relating to patient-centered care [11, 16–18].

The importance of patient involvement was also highlighted in research [19, 20]. While it used to be common practice to involve patients only in the role of research participants, the role of co-researchers is now increasingly coming to the fore [21]. Patients can be involved at various stages of the research process [22, 23]. Involving patients in research brings a number of benefits. Patients can provide valuable insights and knowledge from their personal experiences that might remain hidden from researchers [23]. Through their active involvement, patients can also help to better tailor the study design and research objectives to the needs and preferences of those affected and facilitate the dissemination of research findings [22, 23].

Against this background, patients have become increasingly active partners in patient-provider consultations. Individual characteristics such as gender, age, level of education, locus of control, or character traits play a role in relation to the respective preferences and desires of patients [24–31]. Patients’ preferences for the decision-making process may be ascertained following the notion of Emanuel & Emanuel [1] who define different models of decision making: a paternalistic model, an informative model, an interpretative model and a deliberate model with different roles for doctors and patients. Research has shown that patients have different preferences regarding these models, which also seem to be context-bound and not stable over time [32, 33].

Preferences and desires are two closely related concepts. Both are states connected with a wish or intention to do something that determine our behavior [32]. A desire is typically directed at one intentional object and has to be understood as involving a degree of intensity, e.g. patients’ desire for autonomy [34]. Preferences are typically bi-directional, for example a doctor-oriented versus a patient-oriented style of communication [13, 24, 33].

Preferences on the provision of healthcare can be assessed in various quantitative ways, like ranking, rating or choice-based approaches as well as with qualitative techniques like either individual or group-based approaches. Typical techniques to measure consumer preferences were developed in the context of market research and contain discrete choice experiments, best–worst scaling methods, willingness to pay or standard gamble methods [35, 36]. However, no single method fits for all purposes and methods and therefore needs to be chosen carefully [35].

In addition to a research perspective about how to elicit patient preferences and desires in research projects to inform practice, tools and instruments are needed to elicit patient preferences in daily clinical practice. This would allow for more individualized, patient-centered and effective care and would increase patient satisfaction as well as quality of care [7, 37–47]. Considering that not all patients want to be involved in decisions [48], studies also explored the meaning of congruence between providers and patients in relation to patient-centeredness and patient involvement [24, 49, 50].

Policy recommendations, communication strategies and easy-to-handle questionnaires are therefore required to measure patients’ preferences so that health care providers can be encouraged to investigate such preferences in daily clinical patient consultations [41]. A variety of questionnaires have been developed to adequately assess patients' preferences with regard to communication, participation, information needs, and treatment decision involvement, although these instruments were often developed for research purposes rather than for regular use in clinical settings. However, an overview of these instruments is still lacking and makes it difficult for clinicians to identify suitable instruments for eliciting patient preferences and desires appropriately in different clinical contexts. It is also not clear which instruments have been jointly developed with patients using participatory research approached [51–53] to ensure that patients’ experiences and expectations have been sufficiently covered. The aim of this scoping review was therefore to provide a systematic overview of the existing questionnaires with regard to content, structure, and development process including the involvement of patients.

## Methods

We used a scoping review approach because we aimed to map an area of available studies, i.e. existing questionnaires to elicit patient preferences, and to synthesize research to provide an overview of tools. The focus was not on assessing the quality of the studies, as would be typical for systematic reviews. The scoping review was conducted in a five-stage approach following Arksey and O'Malley [54]: 1) formulating the research question 2) identifying relevant studies 3) selecting studies 4) collecting the data 5) collating, summarizing and reporting the results. The process was dynamic and iterative, and followed the PRISMA extension for scoping reviews [55]. Each step was discussed with the research group and recorded in a protocol as part of the quality assurance process.

### Formulation of the research questions

We aimed to answer the following research question:

Which questionnaires can be used to elicit patients' preferences regarding patient-provider consultations?

This led to the following sub-questions:

1. How have questionnaires been developed to capture patient preferences regarding information, decision-making, communication, and involvement in patient-provider-consultations?
2. To what extent have patients been involved in the development of instruments?
3. Which dimensions and categories have been covered?
4. What is the structure of instruments in terms of length, prompting and response format of items?
5. What is the internal consistency and/or reliability of published instruments?

### Identification of relevant studies

The search strategy was developed by AB and CK and subsequently reviewed by YYA. The search strategy included four categories of keywords: 1) “patient preference” and synonyms 2) “instrument” and synonyms, 3) “decision making”/“information”, 4) “chronic disease”/“chronic illness” (see Appendix A for the search strings used in PubMed). The keywords were identified through an initial pilot literature search in PubMed and Google Scholar and a snowball search to identify existing instruments and questionnaires. Further instruments were identified through the work of Chewning et al. [2] and Simon et al. [56] who focused on overviews of instruments measuring shared decision making.

The systematic search was then conducted in PubMed and PsycINFO on December 8th, 2022 and then updated on April 5th, 2023. The aim was to identify articles on development, validation, and/or modification of instruments for measuring patients' preferences with regard to information, decision making, involvement, or communication. Inclusion criteria were studies providing primary data of questionnaires, which were published in German or English, and included adult patients with a chronic disease as the study population. Translations of instruments were included when the original instrument was adapted, modified or shortened while translated. Exclusion criteria were defined as follows: articles that did not describe a questionnaire (e.g. discrete choice experiments not using questionnaires but others forms of measurement [36], card sort sets [3]) or used questionnaires already developed; translation of instruments without any modifications of the original instrument; studies with the study population of children and adolescents; acute illnesses and emergency situations; instruments which did not measure patient preferences but other constructs such as quality of life, evaluation of providers’ behaviors, or experience of chronic illnesses; and articles presenting secondary data. Studies using a healthy population to pilot a questionnaire which could be used in a population of patients with chronic diseases were not excluded.

No article was excluded due to insufficient psychometric data. If an instrument was described in more than one article, we tried to identify the first publication as we were mainly interested in the structure of the instruments and the development process which both is typically best described in early publications of instruments. A second snowball search was conducted and all articles that met the inclusion criteria were included. If authors referred to a previously published instrument, the article of the first description was searched for and included if inclusion criteria were met. The search strategy was also discussed and modified with PB, DW, and YYA. Rayyan [57] was used to collect and organize the articles. Decisions were documented in a study protocol.

### Study selection

All titles and abstracts of the literature search were imported into the Rayyan software and duplicates were removed. To ease collaboration between the reviewers, Rayyan was used allowing the reviewers to review articles in parallel. Other features offered by Rayyan, such as an AI-assisted study selection, were not employed. For calibration, 500 abstracts were assessed in a first round and 400 abstracts in a second round in a blinded manner by two researchers (AB and CK). Studies were categorized as "include", "exclude" and "maybe". The results were then unblinded, conflicts and "maybe" decisions were subsequently discussed in the research team and assigned to the "include" or "exclude" category by consensus. As a high degree of consensus was achieved in the second calibration process, remaining abstracts were divided among AB and CK, and assessed independently. Additional “maybe” decisions were resolved in regular team meetings.

Full text articles were imported into Endnote and screened for eligibility using the same inclusion and exclusion criteria as for the abstracts. For calibration, the first ten articles were screened in parallel by AB, DW and CK. Conflicts were discussed and resolved by consensus. The remaining full text articles were screened by AB and DW. In case of ambiguity, articles were discussed and decided upon in research team meetings.

### Data extraction

Data of the included studies were extracted using a data extraction sheet with the following information: First author, title, publication year, country, name of instrument, aim of study, setting/recruitment, patient population, sample size of study population, development process of instrument, theoretical construct/ topic, number of items, structure of instrument (type, prompt, response format), reliability/internal consistency (Cronbach's α; KR 20; test–retest), other psychometric information (see Appendix B). Data extraction was piloted with five articles and discussed in the research team. First results were discussed by the team. The data extraction sheet and study protocol were refined afterwards. Data was extracted by AB and DW, reviewed by CK in regular meetings, and then agreed in joint discussions.

### Compiling, summarizing and reporting the results

We obtained mainly quantitative results from the data extraction sheet. Data were synthesized according to the process of instrument development defined by Kalkbrenner [58]. Different approaches of patient involvement were categorized according to van Overbeeke et al., [59] and Williamson et al. [22].

The results are reported below in tabular (complete data see Appendix B) and narrative forms.

## Results

### Description of included studies

**Fig. 1:**
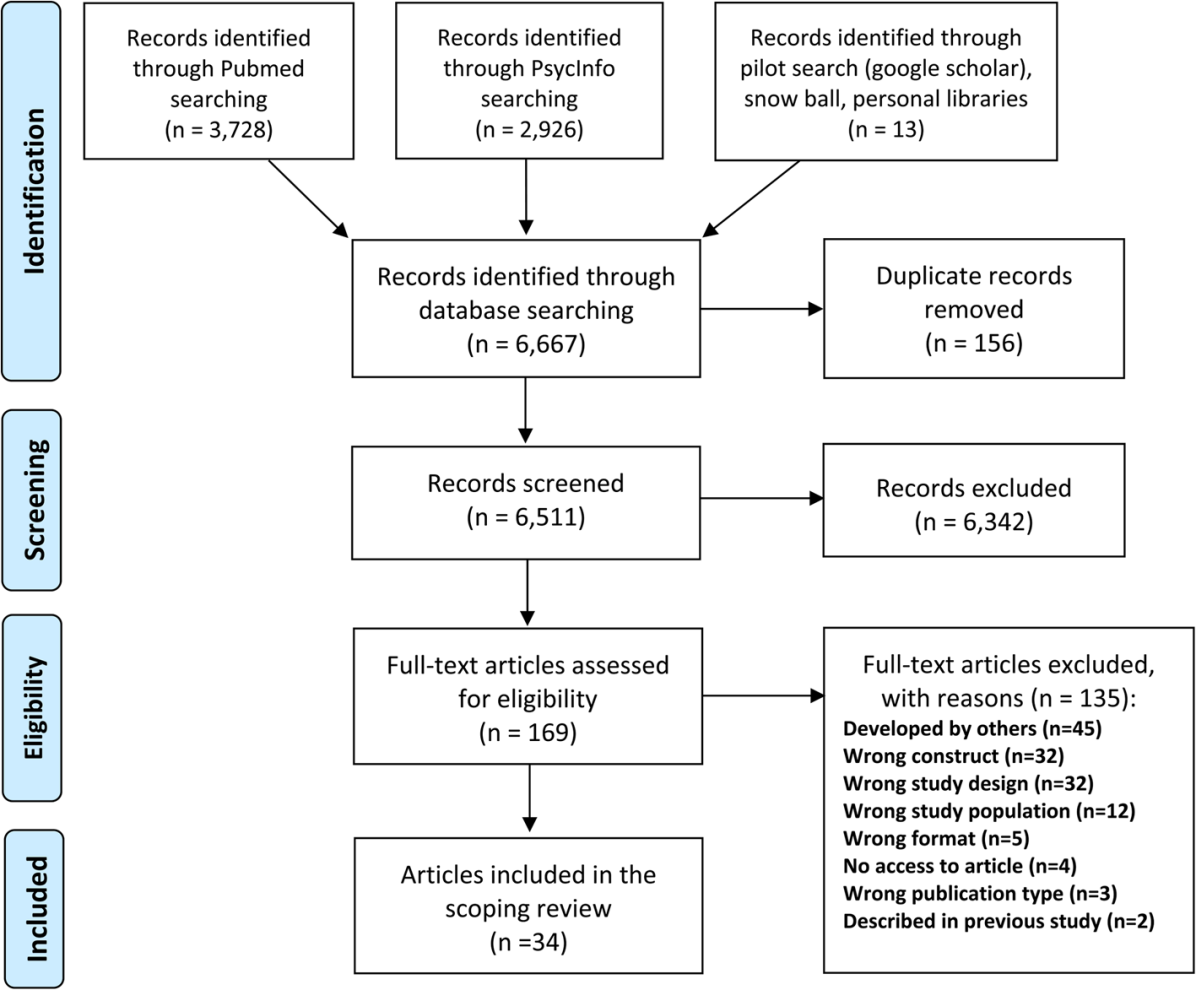
Flow chart scoping review patient preferences instruments

We included 34 [24, 34, 48, 60–90] articles comprising 37 different instruments (see Fig. 1). Adapted versions or adapted translations were included for: Autonomy Preference Index (API) [34, 87], Health Opinion Survey (HOS) [76, 79], Patient–Practitioner Orientation Scale (PPOS) [24, 74, 84], and Ask3Questions [78]. Of the included articles, eight were published between 1980 and 2000 [24, 34, 62, 75, 76, 79, 88, 89], 11 were published between 2001 and 2010 [48, 60, 61, 65, 66, 68, 69, 82, 86, 87, 90], and the remaining 15 articles were published between 2011 and 2021 [63, 64, 67, 70–74, 77, 78, 80, 81, 83–85].

The articles described projects from ten countries: most studies (*n* = 15) originated in the United States [24, 34, 48, 61, 62, 65, 68–72, 76, 81, 88, 90], followed by the United Kingdom (*n* = 6; [60, 66, 67, 79, 82, 86]), Germany (*n* = 5; [64, 78, 83, 84, 87]), Canada (*n* = 3; [63, 75, 89]), one article each from Israel [80], and Switzerland [77]. Three articles described studies conducted in more than one country, including Italy, Denmark, Hungary, and Argentina [73, 74, 85].

### Study population and setting

A large number of studies (*n* = 12) included patients with cancer as study population [60–63, 67, 69, 71, 78, 79, 81, 83, 89]. Settings for data collections ranged from national cancer centers [61] through cancer units [67], inpatient and outpatient clinics [78], and ambulatory care facilities [89]. Patients with one specific disease or symptom other than cancer varied from patients undergoing angiogram [75], patients with chronic pain [87], anxiety disorders [77], asthma [86], or hypertension [88]. Eight studies included patients with a variety of diseases [24, 64, 66, 70, 72, 73, 82, 85], e.g. chronic back pain and chronic ischemic heart disease [64], primary care patients [24, 70], mental illnesses [85], advanced life-threatening illnesses [82], rheumatoid arthritis or type 2 diabetes [66], hospitalized patients with multiple comorbidities [72], with COPD or cancer [73]. Study settings were university centers, hospitals, rehabilitation clinics, outpatient and primary care clinics, or hospices. Some studies included students [74–76, 84, 90], health professionals [24, 82, 85] or healthy adults [48, 80, 90], high school graduates from a Longitudinal study [65], or African American men recruited in urban barber shops [68]. Recruitment of study population also included large scale recruitment settings [68, 71].

All in all, 27 studies included patients. Sample size of included patient population ranged from 34 [78] to 1.690 [81], in total 10.465 patients, mean 371 patients. 13 studies included other study populations, clinicians or other health professionals [24, 69, 82, 85, 88], students [74–76, 84, 90], and healthy/general population [48, 65, 68, 75, 80, 90]. Total sample size was 15,780, ranging from 10 [75] to 10,317 [65] with a mean of 1,015.

### Development process of instruments

Most articles described the development process of the instruments, three articles did not provide information about the development process [24, 81, 88]. Granularity, topics, and focus of the development process varied considerably. Whereas few authors provided detailed information [64, 74, 76, 78, 85], most descriptions of the development process were quite short. Common approaches for the development process were review of literature, selection of items or adaption of items based on an instrument published elsewhere, interviews with patients or other study populations, pilot-testing with patients or other study populations, psychometric analyses, and different ways of reviewing and discussing study results. Of the seven articles describing the adaptation or translation with adaptions of an existing instrument, only few authors provided detailed information about the translation and adaptation process [74, 78] (see Table 1).

**Table 1:** Description of development phases of questionnaires

Description of phases of development process	Articles
Review of literature, theoretical models, guidelines etc	*n* = 8 [48, 61, 64, 71, 72, 77, 79, 90]
Selection of items or adaption of an instrument published elsewhere	*n* = 8 [63, 66, 72, 74, 76, 82, 84, 89]
Interviews with patients	*n* = 6 [60, 64, 67, 72, 83, 86]
Interviews with others (providers, students, householders, study population unnamed)	*n* = 4 [60, 68, 80, 90]
Group discussions with patients	*n* = 2 [64, 89]
Development of items based on patient stories or experiences	*n* = 2 [70, 72]
Pilot testing with patients	*n* = 6 [34, 62, 64, 69, 70, 73]
Pilot testing with others (providers, students, households, study population not specified)	*n* = 9 [48, 67, 69, 70, 74–76, 80, 90]
Modified Delphi method	*n* = 1 [34]
Psychometric analysis	*n* = 6 [67, 70, 71, 74, 76, 90]
Review, discussion, consensus forming of results	*n* = 9 [34, 48, 60, 64, 67, 71, 75, 80, 86]
Team translation protocol TRAPD (Translation, Review, Adjudication, Pretesting, Documentation)	*n* = 1 [78]

Twelve studies reported patient involvement into the development of an instrument, mostly in interviews, group discussions, pilot testing, or using patient narratives [34, 60, 62, 64, 67, 69, 70, 72, 73, 83, 86, 89]. Studies did not report patient involvement in defining research questions, sample definition or participant recruitment, research method selection, data collection, analysis or interpretation.

### Description of instruments

#### Themes of instruments

The majority of instruments aim to measure different aspects of information preferences and/or decision making preferences (*n* = 22). Some of the instruments focus on preferences of specific patient groups, mainly cancer patients [60, 63, 69, 79, 83] or specific aspects of information provision and decision making e.g. on medication [66], surgery decision [69], or active surveillance [63]. Fewer instruments (*n* = 5) focus on patient centeredness or communication preferences [24, 64, 74, 80, 84] or aspects of health service or care (*n* = 5), partly in combination with information or decision making preferences [67, 70–72, 86] (see Table 2). Authors most often used the word “preferences” to describe the theoretical construct of their tool (*n* = 20 [48, 62–66, 68, 69, 72, 73, 75, 76, 80–82, 85–88, 90]). Other states were less frequently used, like “needs” (*n* = 5 [61, 67, 77, 79, 83]), “attitudes” (*n* = 2 [60, 84]), “desires” (*n* = 2 [34, 89]), “engagement/empowerment” (*n* = 2 [71, 78]), “orientation” (*n* = 2 [34, 74]), or “priorities” (*n* = 1 [70]).

**Table 2:** Themes of questionnaires

Theme of questionnaire	Name of questionnaire (no. of items) [Reference]
**Decision making preferences**	
Preferences in decision-making	Ask 3 Questions (3 items) [78]
Cancer patients’ attitudes towards their involvement in decision making	Attitude rating scale (26 items) [60]
Patient preferences for decision making involvement of medication	Control preference scale [3], adapted version (CPS) (3 items in addition to CPS) [66]
Women’s preferred role in the breast surgery decision	Control preference scale [3], adapted version (CPS) (1 item in addition to CPS) [69]
Degree of control an individual desired in the decision-making process	Preference for control in decision making (1 item) [68]
Patient perceptions of autonomy-supportive communication from their surgeons and oncologists	No name of instrument provided by authors (2 items) [81]
**Information preferences**	
Information needs among cancer patients	Cancer Patients Information Needs (CaPIN) (23 items) [83]
Priority information needs of women newly diagnosed with breast cancer	No name of instrument provided by authors (9 items) [79]
**Decision making preferences and information preferences**	
Patients' preferences for two dimensions of autonomy: decision making and information seeking	Autonomy Preference Index (API) (23 items) [34]
Patients' preferences for two dimensions of autonomy: decision making and information seeking	Autonomy Preference Index; German version (API) (14 items) [87]
Patients' preferences for two dimensions of autonomy: decision making and information seeking for use in mental health care	Clinical Decision Making Style Scale (CDMS) (21 items) [85]
Preferences for health information and participation in health decision-making	Health Information Wants Questionnaire (HIW) (80 items) [90]
Preferences for different treatment approaches: Decision making preferences; preferences for information, and behavioral involvement in medical care	Health Opinion Survey (HOS) (16 items) [76]
Patients’ preferences for information needs and involvement in treatment decisions	Health Opinion Survey (HOS) (16 items), Information Seeking Questionnaire (ISQ) (18 items), Preference for Participation in Treatment Decisions Questionnaire (5 items) [89]
Degree to which patients prefer to become informed about and to participate in their medical care	Information Styles Questionnaire (15 items) [62]
Patient preferences for involvement in treatment decision making and desire for information	Problem Solving Decision-Making Scale (PSDM) (18 items) [75]
Information and decision making preferences of patients with prostate cancer on active surveillance	No name of instrument provided by authors (7 items relevant to this review) [63]
Health care decision making preferences: information exchange, deliberation, and selection of treatment choice	No name of instrument provided by authors (4 items relevant to this review) [65]
Patients’ preferences for illness information and treatment decision making	No name of instrument provided by authors (30 items) [73]
Public preferences for participation	No name of instrument provided by authors (3 items) [48]
Patients’ information and decision-making needs as a prerequisite for the development of patient decision aids for anxiety disorders	No name of instrument provided by authors (30 items) [77]
Patient preferences for information and involvement in decision making as a clinical tool	No name of instrument provided by authors (8 items) [82]
Patients’ information preference about their disease and its therapy and preferred involvement in treatment decisions	No name of instrument provided by authors (4 items) [88]
**Preferences for patient centeredness, communication styles, or aspects of provider-patient relationships**	
Communication preferences of chronically ill patients	Communication preferences of patients with chronic illness (KOPRA questionnaire) (32 items) [64]
Patient-centeredness of physicians and patients with the two dimensions sharing and caring	Patient–Practitioner Orientation Scale (PPOS) (18 items) [24]
Patient-centeredness of physicians and patients with the two dimensions sharing and caring	Patient-Provider Orientation Scale; adapted German version (PPOS-D12) (12 items) [74]
Patient-centeredness of physicians and patients with the two dimensions sharing and caring	Patient-Practitioner-Orientation-Scale; adapted German version (PPOS-D6) (6 items) [84]
Relation between trust and shared decision making, and preferred terminology	No name of instrument provided by authors (2 items) [80]
**Information preferences, decision making preferences and/or communication preferences**	
Patients’ informational needs, decision-making preferences, and communication preferences	Communication Preferences for content & format (CPCF) (44 items; 26 relevant to this review) [61]
**Clinical care and service in general, and additional themes**	
Patient experiences or patient needs of chronic cancer: clinical service, self-care and self-management, needs for independent living, work, finances and benefits, psychological experiences, support pathways	Chronic Cancer Experiences Questionnaire (CCEQ) (75 items) [67]
Engagement in decision making in cancer care: Diagnostic acceptance (emotional process), empowerment, involvement (relational processes), information-seeking, planning (cognitive processes)	Decisional Engagement Scale (DES-10) (10 items) & short version (DES-3) (3 items) [71]
Patients' experience and priorities in domains through which they routinely experience their healthcare providers: patient-physician relationships, patients’ personal responsibility for health, diagnostic tests, patient preferences regarding medications, healthcare costs	What matters to you survey (5 items) [70]
Patient preferences for engagement in healthcare used by nurses: information gathering, self-advocacy, informed decision-making, family involvement, active participation, resources	Patient Preferences for Engagement Tool (PPET) (27 items) [72]
Preferences of patients with asthma for attributes or characteristics associated with treatment for their asthma: Extent to active listening, Extent to treatment relief, Chance of experiencing side effects, Extent to continuity of doctors during treatment, Extent to patient centeredness, Travel costs	No name of instrument provided by authors (17 items) [86]

#### Structure of instruments

The length of instruments varies between one item [68, 69] and 80 items [90]. Seventeen instruments contain up to ten items [48, 63, 65, 66, 68–71, 78–82, 84, 88, 89], nine instruments between 11 and 20 items [24, 62, 74–76, 86, 87, 89], and 11 instruments more than 20 items [34, 60, 61, 64, 67, 72, 73, 77, 83, 85, 90]. Short instruments are partly used in combination with other instruments, e.g. with the Control Preference Scale (CPS) [3], which follows a card sort approach and was therefore excluded from this review [66, 69, 78]. Longer instruments consist of two to 14 subscales [67].

Typical structure of questionnaires was starting with statements or lead-in-questions as prompt and different types of response scales, from dichotomous scales [73, 76, 83, 89] to 10-point scales [71]. Sutherland et al. [89] used a linear analog self-assessment to determine different types of information that a patient would like to have about cancer and its treatment. Response formats also present different types of decision making procedures from which the patient chooses the most appropriate one [73, 90]. A few authors have chosen different approaches. Cassileth et al. [62] developed one instrument section according to a single best choice asking patients to select the statement that best describes their point of view from two alternatives. In another section of the instruments, patients are asked to select the type of information desired by clicking a three-point scale from'absolutely need' to "do not want' this information. Hirpa et al. [70] asked patients to rank various choices in order of importance related to different domains with three to seven choices per theme.

Some authors used case scenarios or vignettes to ask patients to indicate their decision making preferences [34, 66, 75, 80, 85]. Kraetschmer [75] combined a vignette with a response format with different types of decision making procedures from which patients select the best option (1 ='doctor alone'; 2 ='mostly doctor'; 3 ='both equally'; 4 ='mostly me'; 5 ='me alone'). A comparable approach was used by Magnezi et al. [80] and Puschner et al. [85]. Luker et al. [79] used pairwise choices to ask patients to decide which of the information needs had the greater importance. This procedure was repeated for 36 pairs of information. A similar approach was used by Ratcliffe et al. [86] with eight pairwise choices. Only one instrument (‘ask three questions’) provided an open response format [78]. The instrument is presented in form of a postcard with three questions about information helpful for the decision making process. Patients are empowered to formulate their own questions in the following consultations.

#### Quality criteria/ psychometric information: reliability/internal consistency

Ten studies reported Cronbach’s alpha or Kuder Richardsen 20 (KR 20) as indicators of internal consistency/reliability for total scales [24, 34, 61, 71, 74, 76, 83, 84, 89, 90]. In addition, Cronbach’s alpha or Kuder Richardsen 20 (KR 20) were reported for subscales in twelve studies [24, 61, 64, 67, 72, 74, 76, 83–85, 87, 89]. Three studies reported indicators in a summarized form with broad ranges of Cronbach’s α for subscales [67, 75, 90]. 18 studies did not report internal consistency/reliability [48, 60, 62, 63, 65, 66, 68–70, 73, 77–82, 86, 88]. Cronbach's α or KR 20 ranged from Cronbach's α = 0.73 (PPOS-D12) [74] to Cronbach's α = 98 [90]. Details are summarized in Table 3. Additional indicators were also reported like test-test reliability [34, 75, 76], factor analysis [74] or discriminant validity [76].

**Table 3:** Cronbach’s alpha or KR 20 of included questionnaires

Cronbach’s α (or KR 20)	Total scales	Subscales / Subpopulations
<.70	PPOS-D6 [84]	API-German Subscale SDM & Subscale IS (premedication visit) [87] CCEQ subscale Accessing support [67] HOS subscale I &HOS subscale B [89] PPOS-D6 Subscale Sharing & Subscale Caring [84] PPOS-D12 Subscale Sharing & Subscale Caring [74] PPOS Subscale Caring Patient sample [34]
.71—.80	DES-3 [71] DES-10 [71] HOS [89] HOS [76] PPOS [34] PPOS-D12 [74]	API-German Subscale IS (chronic pain clinic) [87] CaPIN Subscale 2 & Subscale 3 [83] HOS Subscale I & Subscale B [76] KOPRA subscale Communication about pers. circumstances [64] PPET Subscale RE, Subscale IG, Subscale SA & Subscale FI [72] PPOS Subscale Sharing Patient sample [34]
.81—.90	API [34] CaPIN [83] ISQ [89]	CPCF subscale Diagnosis/Prognosis & subscale Impact of treatment [61] CaPIN Subscale 1 & Subscale 4 [83] CDMS-P PD (section A & B) Patient version [85] KOPRA Subscale Effective and open communication & Subscale Emotionally supportive communication [64] PPET Subscale IDM & Subscale AP [72]
>.91	CPCF [61] HIW [90]	CPCF subscale Treatment Options [61] KOPRA subscale Patient participation and patient orientation [64]

According to Streiner [91], Cronbach's alpha levels of.70 may be acceptable for early stages of research,.80 and above for basic research tools, αs above.90 may indicate redundancy rather than a desirable level of internal consistency

## Discussion and conclusion

### Discussion

We conducted a scoping review to collate information about instruments which explore patients’ preferences regarding information provision, participation in decision making, and patient-provider communication. We aimed to collect information about the development process, the topic and structure, and basic psychometric properties of instruments. In total, we were able to identify 34 articles describing 37 different instruments, the majority of which were published by Anglo-American authors. No articles came from authors located in Asia, Africa, or Australia. Due to the context-bound character of social interaction and communication [92–94], it seems arguable that instruments mainly come from an Angelo-American-European cultural region. Patient preferences regarding provider-patient consultations might differ in different cultural contexts [95] and therefore more research would be needed about patient preferences including specific instruments addressing this context-bound aspect.

A third of the included instruments have been developed or pilot tested with patients with cancer. This also raises the question of the transferability to other patient groups. Patients with cancer might be one of the most vulnerable groups of patients with chronic diseases facing life-threatening diagnoses. Sufficient information provision and involvement in treatment decisions have been identified as crucial for cancer treatment outcomes and patient satisfaction [96–98]. Although other patient groups have been studied [28, 41, 42], an expansion of the spectrum of diseases would certainly be desirable. Due to the inclusion and exclusion criteria of this scoping review, it is important to note that we did not include all studies that examined patient preferences using questionnaires, but only studies reporting on the development of instruments. It would therefore be reasonable to include further patient groups into follow-up studies.

As participatory research has become more and more important in recent years [51–53], we evaluated the extent to which patients were included in the development process of instruments. Studies reported mainly interviews, group discussions, and pilot testing with patients. However, only a third of the articles reported these approaches. Two articles reported the development of items based on patient narratives [70, 72]. Further approaches to involve patients into the research process like patient boards or patients as co-researchers were not reported. Involvement focused on the design of instruments, rather than other stages of the research process, such as defining research questions, designing study methods, data collection, analysis or interpretation. However, especially with a topic such as patient preferences, these could be interesting approaches to address topics that are not anticipated by researchers because they do not have the life-world experience of patients. Important insights gathered from patient involvement could certainly elucidate new aspects here in the future.

Another finding was that the majority of instruments focusing on different aspects of information and/or decision-making preferences. Patient preferences and expectations regarding provider-patient communication or organizational aspects of patient consultations were less common. The dominance of instruments focusing on shared-decision making and information provision may be due to the increasing relevance of shared-decision models over the last 25 years, which has led to an increasing number of research projects and publications [99]. However, since patient centeredness and the care of patients include a number of other process steps in a consultation, e.g. initiating a session, gathering information as part of the medical history, advice on changes in lifestyle or cross-sector and cross-professional support during challenging phases of illness [100–103], it would be desirable to pay more attention to these process steps as well and to further explore patients' preferences and wishes.

The granularity and specificity of included questionnaires varied immensely. Instruments contain between one and 80 items. Longer questionnaires contain up to 14 subscales. A variety of instruments allow to select from already published instruments for very specific study purposes. This scoping review focused on articles describing the development, validation and initial implementation of instruments. This may explain why most of the instruments were used for research purposes. Only one article explicitly stated that the primary purpose of the development of the instrument was to be used in real clinical settings [78]. However, our findings do not allow us to recommend one tool over another. In addition, the description of instruments varied immensely and in some cases the reported information made it difficult to understand the scope, structure and psychometric properties of instruments.

Only a third of the articles reported Cronbach’s alpha or Kuder Richardsen 20 (KR 20) as indicators of internal consistency/reliability for total scales. A more consistent reporting of the development of instrument, scope, structure, and psychometric properties would be desirable for future publications. Also rarely reported for the instruments was a dedicated measurement model– examined by means of a hypothesis-testing framework such as confirmatory factor analysis. While it is reasonable to assume that follow-up studies conducted on the instruments– such as studies on adaptations to other languages or populations– employ such approaches, the indications are that the development process of questionnaires may have lacked a thorough theoretical underpinning. The MEASURE approach established by Kalkbrenner in 2021 [58] was helpful for us to structure the data extraction and reporting. For future publications reporting on the development or validation of a new instrument, this guideline may be helpful in checking for completion of relevant information of the development process.

### Limitation

Our scoping review focused on articles about the development of questionnaires to measure patients’ preferences regarding provider-patient communication, information provision, and decision making in English and German. As these topics are characterised by a wide variety of terms and concepts, we may have missed some instruments due to our selection of key words. We have taken great care to identify relevant key words through pilot searches and key articles. At the same time, it was necessary to narrow down the search. Due to the inclusion and exclusion criteria, we may have missed instruments in other languages. Against the background of the context-relatedness of social interaction, further reviews seem necessary to address instruments from Asia, Africa, and Middle- and South America with a broader variety of languages. Some questionnaires have been used in many different studies for very specific purposes. These were not included into our review as we focused on the development and structure of instruments. So there may be further validation studies or studies with other study populations using specific instruments. If readers are interested to measure patient preferences in a specific setting, this review might be a good starting point. Further articles not included in this review need to be studied for additional information of specific instruments. The last update of our literature search was conducted in April 2023. Since then, newly developed questionnaires might have been published. Our review did not address the question of whether questionnaires were or should be presented in paper-and-pencil versions or as a digital tool. When mentioned in the included articles, both online surveys (e.g. [71, 77]) and paper-and-pencil versions (e.g. [60, 74, 78]) were used. New technologies will undoubtedly create new opportunities for the delivery and dissemination of questionnaires to patients and providers. Further research is needed to explore the advantages and disadvantages of these potential new developments.

### Conclusion

Our scoping review revealed a broad variety of questionnaires measuring patients’ preferences related to provider-patient communication, information provision, and decision making. Instruments vary considerably on topics, structure and length, which facilitates the choice of a specific instrument for specific purposes. However, instruments focusing on decision making preferences and information provision in specific settings were predominant. As many instruments have been developed in an Anglo-American context, and patient preferences may differ in different cultural contexts, more research on patient preferences would be needed, including specific instruments addressing this context-bound aspect. Our impression was that questionnaires were mainly developed for research purposes like researching patient preferences of specific patient groups, on specific topics, or in specific settings. The extent to which these questionnaires can also be used in daily clinical care routines remains to be seen. Further research is needed into the practical application of measuring instruments in everyday clinical practice.

### Practice implications

The consideration of patient preferences is now of great importance in the field of health research. A large number of instruments have been published that make it possible to evaluate the preferences of different patient groups for specific topics in certain care settings. Further research is needed to determine how these research findings can be applied more widely in everyday clinical care, and what options or instruments are available to appropriately elicit patient preferences in everyday clinical practice and incorporate them into the care process.

## Supplementary Information

Supplementary Material 1.

Supplementary Material 2.

Supplementary Material 3.

## Data Availability

All data generated or analyzed during this study are included in this published article and its supplementary information.
