# Correction: Which questionnaires can be used to elicit patients’ preferences regarding patient-provider consultations? Results of a scoping review

**DOI:** 10.1186/s12913-025-13553-4

**Published:** 2025-10-07

**Authors:** Alina Zoe Bambas, Diana Wahidie, Yüce Yilmaz-Aslan, Patrick Brzoska, Claudia Kiessling

**Affiliations:** 1 Education of Personal and Interpersonal Competencies in Health Care, Faculty of Health, Witten/Herdecke University, Alfred-Herrhausen-Str. 50, Witten, 58455 Germany; 2 Department Health Services Research, Faculty of Health, Witten/Herdecke University, Witten, Germany

**Correction to**: ***BMC Health Services Research*** **(2025) 25:502. **10.1186/s12913-025-12567-2

Following publication of the article, the authors reported an error in ‘Quality criteria/ psychometric information: reliability/ internal consistency’ subsection and in Table 3. They have mistakenly stated that Pauli et al. had not provided full values for the psychometric indicators of the PPOS-D6 scale. The correct and incorrect text in the ‘Quality criteria/ psychometric information: reliability/ internal consistency’ subsection and the incorrect and correct version of Table 3 are included below and the original article has been corrected.

Original (incorrect) text in the ‘Quality criteria/ psychometric information: reliability/ internal consistency’ subsection:

Nine studies reported Cronbach’s alpha or Kuder Richardsen 20 (KR 20) as indicators of internal consistency/reliability for total scales [24, 34, 61, 71, 74, 76, 83, 89, 90]. In addition, Cronbach’s alpha or Kuder Richardsen 20 (KR 20) were reported for subscales in eleven studies [24, 61, 64, 67, 72, 74, 76, 83, 85, 87, 89]. Pauli et al. [85] only described indicators qualitatively as being poor without presenting exact values (PPOS-D6). Three studies reported indicators in a summarized form with broad ranges of Cronbach’s α for subscales [67, 75, 90]. 18 studies did not report internal consistency/reliability [48, 60, 62, 63, 65, 66, 68–70, 73, 77–82, 86, 88]. Cronbach’s α or KR 20 ranged from Cronbach’s α = 0.73 (PPOS-D12) [74] to Cronbach’s α = 98 [90]. Details are summarized in Table 3. Additional indicators were also reported like test-test reliability [34, 75, 76], factor analysis [74] or discriminant validity [76].

Correct text in the ‘Quality criteria/ psychometric information: reliability/ internal consistency’ subsection (changes marked in bold):

**Ten** studies reported Cronbach’s alpha or Kuder Richardsen 20 (KR 20) as indicators of internal consistency/reliability for total scales [24, 34, 61, 71, 74, 76, 83, **84**, 89, 90]. In addition, Cronbach’s alpha or Kuder Richardsen 20 (KR 20) were reported for subscales in **twelve** studies [24, 61, 64, 67, 72, 74, 76, 83, **84**, 85, 87, 89]. Three studies reported indicators in a summarized form with broad ranges of Cronbach’s α for subscales [67, 75, 90]. 18 studies did not report internal consistency/reliability [48, 60, 62, 63, 65, 66, 68–70, 73, 77–82, 86, 88]. Cronbach’s α or KR 20 ranged from Cronbach’s α = 0.73 (PPOS-D12) [74] to Cronbach’s α = 98 [90]. Details are summarized in Table 3. Additional indicators were also reported like test-test reliability [34, 75, 76], factor analysis [74] or discriminant validity [76].

Original (incorrect) Table 3:

**Table 3:** Cronbach’s alpha or KR 20 of included questionnaires

Cronbach’s α (or KR 20)	Total scales	Subscales / Subpopulations
< .70	-	API-German Subscale SDM & Subscale IS (premedication visit) [87]
		CCEQ subscale Accessing support [67]
		HOS subscale I &HOS subscale B [89]
		PPOS-D12 Subscale Sharing & Subscale Caring [74]
		PPOS Subscale Caring Patient sample [34]
.71 − .80	DES-3 [71]	API-German Subscale IS (chronic pain clinic) [87]
	DES-10 [71]	CaPIN Subscale 2 & Subscale 3 [83]
	HOS [89]	HOS Subscale I & Subscale B [76]
	HOS [76]	KOPRA subscale Communication about pers. circumstances [64]
	PPOS [34]	PPET Subscale RE, Subscale IG, Subscale SA & Subscale FI [72]
	PPOS-D12 [74]	PPOS Subscale Sharing Patient sample [34]
.81 − .90	API [34]	CPCF subscale Diagnosis/Prognosis & subscale Impact of treatment [61]
	CaPIN [83]	CaPIN Subscale 1 & Subscale 4 [83]
	ISQ [89]	CDMS-P PD (section A & B) Patient version [85]
		KOPRA Subscale Effective and open communication & Subscale Emotionally supportive communication [64]
		PPET Subscale IDM & Subscale AP [72]
> .91	CPCF [61]	CPCF subscale Treatment Options [61]
	HIW (90)	KOPRA subscale Patient participation and patient orientation [64]

According to Streiner [91], Cronbach’s alpha levels of 0.70 may be acceptable for early stages of research, 0.80 and above for basic research tools, αs above 0.90 may indicate redundancy rather than a desirable level of internal consistency

Correct Table 3 (changes marked in bold):

Table 3: Cronbach’s alpha or KR 20 of included questionnaires.

**Table:** 

Cronbach’s α (or KR 20)	Total scales	Subscales / Subpopulations
< .70	**PPOS-D6 [84]**	API-German Subscale SDM & Subscale IS (premedication visit) [87]
		CCEQ subscale Accessing support [67]
		HOS subscale I &HOS subscale B [89]
		**PPOS-D6 Subscale Sharing & Subscale Caring [84]**
		PPOS-D12 Subscale Sharing & Subscale Caring [74]
		PPOS Subscale Caring Patient sample [34]
.71 − .80	DES-3 [71]	API-German Subscale IS (chronic pain clinic) [87]
	DES-10 [71]	CaPIN Subscale 2 & Subscale 3 [83]
	HOS [89]	HOS Subscale I & Subscale B [76]
	HOS [76]	KOPRA subscale Communication about pers. circumstances [64]
	PPOS [34]	PPET Subscale RE, Subscale IG, Subscale SA & Subscale FI [72]
	PPOS-D12 [74]	PPOS Subscale Sharing Patient sample [34]
.81 − .90	API [34]	CPCF subscale Diagnosis/Prognosis & subscale Impact of treatment [61]
	CaPIN [83]	CaPIN Subscale 1 & Subscale 4 [83]
	ISQ [89]	CDMS-P PD (section A & B) Patient version [85]
		KOPRA Subscale Effective and open communication & Subscale Emotionally supportive communication [64]
		PPET Subscale IDM & Subscale AP [72]
> .91	CPCF [61]	CPCF subscale Treatment Options [61]
	HIW (90)	KOPRA subscale Patient participation and patient orientation [64]

According to Streiner [91], Cronbach’s alpha levels of 0.70 may be acceptable for early stages of research, 0.80 and above for basic research tools, αs above 0.90 may indicate redundancy rather than a desirable level of internal consistency

