# Supplementary material for: Which questionnaires can be used to elicit patients’ preferences regarding patient-provider consultations? Results of a scoping review
Source: BMC Health Serv Res. 2025 Apr 4;25:502. doi: 10.1186/s12913-025-12567-2 (PMC11971755; doi:10.1186/s12913-025-12567-2)
Supplement: Supplementary file 1 — Supplementary Material 1. [file 12913_2025_12567_MOESM1_ESM.docx]

**Appendix A: Search Strategy for PubMed**

PubMed patient preference* [Title/Abstract] OR patient expectation* [Title/Abstract] OR patient
need* [Title/Abstract] OR patient participat* [Title/Abstract] OR patient involve* [Title/Abstract]

AND

questionnaire [Title/Abstract] OR instrument [Title/Abstract] OR measure* [Title/Abstract] OR tool [Title/Abstract] OR scal* [Title/Abstract]

AND

decision making [Title/Abstract] OR information [Title/Abstract]

AND

chronic disease [MeSH] OR chronic illness [MeSH]
