# Supplementary material for: Which questionnaires can be used to elicit patients’ preferences regarding patient-provider consultations? Results of a scoping review
Source: BMC Health Serv Res. 2025 Apr 4;25:502. doi: 10.1186/s12913-025-12567-2 (PMC11971755; doi:10.1186/s12913-025-12567-2)
Supplement: Supplementary file 2 — Supplementary Material 2. [file 12913_2025_12567_MOESM2_ESM.pdf]

Appendix B: Which instruments can be used to record patients' preferences regarding information, decision-making, involvement, and communication with health care providers? Results of a scoping review

Name first author	Title	Publication year	Country	Name of instrument	Setting/recruitment	Study population/ patient groups	Sample size patients	Sample size others	Objective of the use of the instrument	Development process of the instrument	Theoretical construct/ topic	Number of items	Structure of instrument (sunscales, prompt, response format)	Reliablity/internal consistency (Cronbach's α; KR 20; test-retest)
Beaver [60]	Colorectal cancer patients attitudes towards involvement in decision making	2009	UK	Attitude rating scale	3 sites: 2 cancer centres and a large district general hospital	Adults with a newly diagnosed colorectal cancer	375		To explore Colorectal cancer patients attitudes towards their involvement in decision making	Based on three stages:<br>(1) in-depth qualitative interviews with patients<br>(2) using in-depth interviews with 35 medical, nursing and allied health professionals.<br>(3) developing an attitude rating scale based on stage 1 and 2	Decision making preferences	Total: 26	3 main sections:<br>1) attitude rating scale (26 statements)<br>2) demographic/treatment details<br>3) one open question to reflect on responses and provide general comments<br>Response format: 5-point scale ranging from 'strongly agree' to 'strongly disagree'<br>For six statements, a not applicable option was necessary	Not reported
Buzaglo [61]	An Internet method to assess cancer patient information needs and enhance doctor-patient communication: A pilot study	2007	USA		National cancer centre	Patiens with advanced cancer	46		To assess patient informational needs and decision-making preferences and to electronically generate a printable, patient summary delivered to the oncologist prior to the initial doctor-patient consultation.	Based on<br>(1) literature review and study aims	Information preferences<br>Decision making preferences<br>Communication preferences for content and format	Total: 44<br>Subscale 1: 26<br>Subscale 2: 18	2 subscales:<br>1) Communication preferences with a) diagnosis/prognosis, b) treatment options, c) the way in which cancer and treatment may affect emotions, work, daily routines and relationships<br>Response format: 5-point scale from 1 = Not at All, to 5 = "Very Much<br>2) Satisfaction with content and format of communication<br>Response format: 5-point scale from 1 = Dissatisfied to 5 = Satisfied	1) Communication preferences for content and format<br>Total scale: Cronbach's α = .93<br>Subscale Diagnosis/Prognosis: Cronbach's α = .83<br>Subscale Treatment Options: Cronbach's α = .96<br>Subscale Impact of Treatment on QOL: Cronbach's α = .85<br>2) Satisfaction with content and format of communication<br>total scale: Cronbach's α = .97<br>Diagnosis/Prognosis: Cronbach's α = .93<br>Treatment Options: Cronbach's α = .92<br>Impact of Treatment on QOL: Cronbach's α = .93
Cassileth [62]	Information and participation preferences among cancer patients	1980	USA	Information Styles Questionnaire	Inpatient & outpatient setting	Patients with cancer	246		To explore the degree to which patients prefer to become informed about and to participate in their medical care	Based on<br>(1) pilot testing with patients	Information preferences<br>Treatment decision making preferences	Total: 15<br>Section 1: 3<br>Section 2: 12	2 sections<br>1) general items on information and decision<br>a) information preferences: prompt not reported; response format: on a five-point scale from 1 = 'no more details than needed' to 5 'as many details as possible'<br>b) Decision making preferences: Prompt: select the statement that best describes their point of view from two alternatives; response format: 'leave decision up to doctor' or 'participate in decisions'<br>c) Information preferences:prompt: select one of the following statements; response format: 'I want only the information needed to care for myself properly.', 'I want additional information only if it is good news' or 'I want as much information as possible, good and bad.'<br>2) Prompt: list of items to determine the type of information desired by patients<br>response format: 3-point scale from 'absolutely need' to "do not want' this information	Not reported
Davison [63]	Factors influencing treatment decision making and information preferences of prostate cancer patients on active surveillance	2012	Canada		Community based urology clinic	Patients with prostate cancer	180		To explore the information and decision making preferences of patients with prostate cancer on active surveillance	Based on<br>(1) results of a qualitative study published elswhere	Information preferences while on active surveillance	Only one part relevant to review<br>Part 3: 7	3 parts; only part 3 relevant for this review<br>3) information preferences while on acitve surveillance<br>amount of informaton wanted in the following areas: eating a 'prostate friendly' diet; learning about other men's experiences about being on AS; use of non-traditional treatments; learning about currently available treatment options; having access to an AS patient support group; having access to an internet based AS patient support group; and participation in an exercise program<br>Response format: 5-point Likert Scale from 1= not at all to 4 = a great deal	Not reported
Ende [24]	Measuring patients' desire for autonomy: decision making and information-seeking preferences among medical patients	1989	USA	Autonomy Preference Index (API)	Hospital-based primary care clinic	Not described in detail	313		To explore patients' preferences for two dimensions of autonomy: decision making and information seeking.	Based on 3 stages<br>(1) Modified Delphi study involving clinicians, medical sociologists, and ethicists, carried through two iterations<br>(2) field test of items with patients to ensure content validity<br>(3) Review of items based on based on patients' feedback from patients and psycometric properties of items and instrument	Information preferences<br>Decision making preferences	Total: 23<br>1. information seeking: 8<br>2. decision making total: 15<br>2A general items: 6<br>2B 3 vignettes with 3 items each	2 parts<br>1. information seeking scale<br>Prompt: statements<br>Reponse format: 5-point Likert scale from 'strongly disagree' to 'strongly agree'<br>2. decision making preference scale<br>A) General items<br>Prompt: statements<br>resonse format: 5-point Likert scale from 'strongly disagree' to 'strongly agree'<br>B) Vignettes.<br>Prompt: case scenario and lead-in question<br>Response format: 3-point scale: 'you alone', 'mostly you', 'the doctor and you'	Internal consistency reliability:<br>Each scale: Cronbach's α = 0.82<br>Retest-Reliability (Pearson product-moment correlations) Decision-making-scale: r = .84<br>Information-seeking-scale: r = .83
Farin [64]	Development of a questionnaire to assess communication preferences of patients with chronic illness	2011	Germany	Communication preferences of patients with chronic illness (KOPRA questionnaire); (Kommunikationspra eferenzen von Patienten mit chronischen Erkrankungen)	Rehabilitation clinic	Patients with chronic back pain or chronic ischemic heart disease	539 (total)<br>472 (survey)<br>57 (focus groups)<br>10 (cognitive interviews)		To identify the communication preferences of chronically ill patients. Clinical use of the questionnaire appears useful to determine patients' communication preferences.	Based on 5 stages<br>(1) First item development based on literature and compilation of relevant aspects of communication with provider to a 'List of Communication Aspects' (version 1)<br>(2) Focus groups to integrate the communication new aspects introduced by patients<br>(3) Item generation based on focus groups and ''List of Communication Aspects'' (Version 2, 105 items)<br>(4) Cognitive interviews with patients; afterwards, comprehension probe; patients' comments were used to improve the item formulation<br>(5) Analysis of item properties based on a survey with patients	Communication preferences regarding clinicians, nurses, therapists:<br>Patient participation and patient orientation<br>Effective and open communication<br>Emotionally supportive communication<br>Communication about personal circumstances	Phyisician version: Total: 32<br>subscale 1: 11<br>subscale 2: 10<br>subscale 3: 6<br>subscale 4: 5	4 subscales<br>1) Patient participation and patient orientation<br>2) Effective and open communication<br>3) Emotionally supportive communication<br>4) Communication about personal circumstances<br>Prompts: statements<br>Resonse format: 5-point scale from 1 = 'not so important' to 5 = 'extremely important'	1) Patient participation and patient orientation: Cronbach's α = .92<br>2) Effective and open communication: Cronbach's α = .89<br>3) Emotionally supportive communication Cronbach's α = .84<br>4) Communication about personal circumstances Cronbach's α = .80
Flynn [65]	Personality and health care decision-making style	2007	USA		High schools (data from the Wisconsin Longitudinal Study (WLS))	High school graduates		10317 (High school graduates)	To exlore relationships between personality factors and health care care decision making preferences	Based on<br>(1) existing data set: data from the Wisconsin Longitudinal Study (WLS) graduate survey, a long-term cohort study of men and women who graduated from Wisconsin high schools in the spring of 1957. All variables in their analysis came from the WLS 2004 mail or telephone survey unless otherwise noted.	Information preferences<br>Treatment decision preferences (deliberation, and selection of treatment choice)	Total: 4	1 scale with 4 items presenting statements about preferences during health care visits for:<br>1) information (2 items: need for history taking, information about treatment options)<br>2) deliberation (discussion of treatment choices)<br>3) decisional control (selection of treatment choice)<br>Instruction: Please think about the doctor that you usually go to when you are sick or need advice about your health and indicate how much you agree or disagree with each statement.<br>Response format: 5-point scale from 1 = agree strongly to 5 = disagree strongly	Not reported
Garfield [66]	Can patients preferences for involvement in decision-making regarding the use of medicines be predicted?	2007	UK	Control preference scale, adated version (CPS)	Outpatient and primary care clinics	Patients with rheumatoid arthritis or type 2 diabetes	516		To explore patient preferences for decision making involvement of medication	Based on<br>(1) previous qualitative research published elsewhere	Involvement in treatment preferences of medication (starting, changing dosage and stopping medication)	Total: 3<br>(in addition to CPS)	3 additional items relating to starting, changing dosage and stopping medication<br>Prompt: short case scenario with lead-in question<br>Response format: 5-Point-Likert scale: from 'You alone' to 'Doctor (or other healthcare professional) alone'	Not reported
Harley [67]	Evaluating the experiences and support needs of people living with chronic cancer: development and initial validation of the Chronic Cancer Experiences Questionnaire (CCEQ)	2016	UK	Chronic Cancer Experiences Questionnaire (CCEQ)	5 cancer units	Patients with chronic cancer	416		To be used as a clinical instrument to assess patient experiences of chronic cancer or to screen for patient needs; also to be used as an outcome measure for evaluating programmes	Based on 4 phases:<br>(1) conceptual framework and item bank' generated questionnaire structure and content based on previous patient interviews<br>(2) face and content validity: clinicians and patients reviewed and suggest amendments to the item bank<br>(3) pilot testing: tested the clarity of items and feasibility of administration.<br>(4) psychometric properties: administered the instrument across multiple cancer units, and psychometric analysis determined subscale structures, internal consistency and construct validity.	Patients' experience and views on 6 themes<br>1: clinical service<br>2: self-care and self-management<br>3: needs for independent living<br>4: work, finances and benefits<br>5: psychological experiences<br>6: support pathways	Total: 75<br>Themes: 6<br>Subscales: 14	6 themes:<br>Theme 1: clinical service<br>6 subscales: Managing appointments, Coordination of care, Information and questions, Making treatment decisions, General practitioner (GP) involvement, Clinical trials<br>Theme 2: self-care and self-management<br>2 subscales: (Symptom experiences), Symptom non-reporting, Key worker<br>Theme 3: needs for independent living<br>2 subscales: Limitations, Sustaining normality<br>Theme 4: work, finances and benefits<br>1 subscale: Financial advice<br>Theme 5: psychological experiences<br>2 subscales: Worries and anxieties, Sharing feelings with others<br>Theme 6: support pathways<br>1 subscale: Accessing support<br>13 items assessing symptom experiences were retained as single items.<br>Prompt: statements<br>Resonse format: 5-point Likert scale from 'strongly agree' to 'strongly disagree'; response option 'not applicable' was included for most items	13 subscales achieved acceptable Cronbach's α (= .71 – .88)<br>1 subscale (accessing support) attained just below threshold (α = .68).
Hart [68]	Health decision-making preferences among African American men recruited from urban barbershops	2009	USA	Preference for control in decision making	Urban barber shops	African American men		40 (men)	To assess the degree of control an individual desired in the decision-making process	Based on<br>(1) qualitative interviews	Involvement preferences for health decisions	Total: 1	Prompt: 'What role does your doctor play or other regular health care provider play in your health decisions?'<br>Response format: single best; select 1 of 5 options from (a) 'I leave all decisions to doctor/health care provider' to (e) I prefer to make final selection about what health care'<br>Responses were collapsed to reflect passive decision-making styles (options a and b), collaborative styles (option c), and active styles (options d and e).	Not reported
Hawley [69]	Factors associated with patient involvement in surgical treatment decision making for breast cancer	2007	USA	Control preference scale, adapted version (CPS)	Data from registries:<br>Area Surveillance, Epidemiology and End Results (SEER)	Patients with breast cancer	1101	277 (clinicians)	To measure women's preferred role in the surgery decision	Based on<br>(1) pilot testing with patients and surgeons	Decision making preferences in relation to breast surgery	Total: 1<br>(in addition to CPS)	Prompt: to choose the response that best fit how they preferred that the surgery decision had been made.<br>Resonse format: from a 'I would have preferred that the surgery decision was made by my doctor(s) with little input from me' to e "I would have preferred to have made the surgery decision with little input from my doctor(s)"<br>Responses were categorized into three levels: (1) too little involvement (2) right amount of involvement; (3) too much involvement in the surgery decision	Not reported
Hirpa [70]	What matters to patients? A timely question for value-based care	2020	USA	What matters to you survey	Ambulatory care setting; gastroenterology subspecialty clinic	Primary care patients, patients from gastroenterology subspecialty clinic	226		To explore patients' experience and priorities in 5 domains through which patients routinely experience their healthcare providers in the outpatient setting	based on<br>(1) selection of 5 domains through which patients routinely experience their healthcare providers<br>(2) consideration of the use of rating versus ranking scales<br>(3) pilot testing with patients<br>(4) Adjustment of survey scales and clarification of instructions based on results from pilot testing	Patient-physician relationships<br>Patients' personal responsibility on health<br>Diagnostic tests<br>Patient preferences regarding medications<br>Healthcare costs	Total: 5	Prompt: Rank in order of importance various choices related to the 5 domains:<br>1. patient-physician relationships: 7 choices from 1 (most important) to 7 (least important)<br>2. patients' personal responsibility on health: 3 choices from 1 (most important) to 3 (least important)<br>3. diagnostic tests: 3 choices from 1 (most important) to 3 (least important)<br>4. patient preferences regarding medications: 5 choices 1 (most important) to 5 (least important)<br>5. healthcare costs: 3 choices from 1 (most important) to 3 (least important).	Not reported
Hoerger [71]	Development and psychometric evaluation of the Decisional Engagement Scale (DES-10): A patient-reported psychosocial survey for quality cancer care	2016	USA	Decisional Engagement Scale (DES-10) & short version DES-3	Recruitment through NIH Clinical and Translational Science (NCATS) Research Match recruitment tool	Patients with cancer	376		To evaluate and improve the quality of cancer care with these brief patient-reported measures	Based on:<br>(1) establishment of a content domain for decisional engagement based on psychosocial theories of health behavior<br>(2) literature review on decisional engagement in cancer care<br>(3) Organisation of five content domains by consensus<br>(4) Development of survey items corresponding to the five identified content domains using the legacy approach (including reviewing the item-level psychometric properties from prior scales; adapting items from existing items)	Decisional engagement:<br>1) Diagnostic acceptance (emotional process)<br>2) Empowerment<br>3) Involvement (relational processes)<br>4) Information-seeking<br>5) Planning (cognitive processes)	Total: 10<br>Short version: 3	5 domains:<br>1) Diagnostic Acceptance<br>2) Empowerment<br>3) Involvement<br>4) Information-Seeking<br>5) Planning<br>Prompt: statements<br>Response format: 10-point scale from (0) Disagree to (10) Agree	DES-10 scale: Cronbach's α = .80<br>DES-3 short version: Cronbach's α = .75.

Jerofke-Owen [72]	Development and psychometric analysis of the Patient Preferences for Engagement Tool	2020	USA	Patient Preferences for Engagement Tool (PPET)	Hospital (medical, surgical and oncology units)	Hospitalized patients with multiple comorbidities, including hypertension, coronary artery disease, cancer, and diabetes	308		To identify patient preferences for engagement in healthcare used by nurses	Based on:<br>(1) review of the literature and patient guidelines<br>(2) qualitative data published elsewhere<br>(3) Interviews with patients<br>(4) development of items based on patient stories	Information Gathering<br>Self-Advocacy<br>Informed Decision-Making<br>Family Involvement<br>Active Participation<br>Resources	Total: 27	6 factors:<br>1) Information Gathering (IG)<br>2) Self-Advocacy (SA)<br>3) Informed Decision-Making (IDM)<br>4) Family Involvement (FI)<br>5) Active Participation (AP)<br>6) Resources (RE)<br>Prompt: statements + open-ended question<br>Response format: 5-point Likert scale from 1 = 'strongly disagree' to 5 = 'strongly agree'	Cronbach's α were >.70 for all 6 subscales<br>Subscale IG: Cronbach's α = .72<br>Subscale SA: Cronbach's α = .75<br>Subscale IDM: Cronbach's α = .85<br>Subscale FI: Cronbach's α = .76<br>Subscale AP: Cronbach's α = .84<br>Subscale RE: Cronbach's α = .72
Jordan [73]	Examining patients preferences for participation in clinical decision-making: the experience in a Latin American chronic obstructive pulmonary disease and cancer outpatient population.	2014	USA, Argentina		Outpatient setting	Patients with COPD or cancer	99 (total)<br>44 (COPD)<br>55 (cancer)		To explore patients preferences for illness information and treatment decision making	Based on:<br>(1) pilot testing with patients	Information preferences<br>Treatment decision preferences	Total: 30 (reported by authorst), 27 (counting related to sections)<br>section 1: 18<br>section 2: 8<br>section 3: 1	3 sections:<br>Section 1: Patient preferences regarding information desired from their doctors<br>Prompt: questions<br>Response format: 3 response choices (I absolutely want this information, would like this information, I do not want this information)<br>Section 2: Patient preferences regarding information desired from their doctors<br>Prompt: statements<br>response format: yes/ no<br>Section 3: Patient preferences regarding treatment-related decision-making<br>Prompt: Statement completed by set of response options (Treatment decisions should be made by...)<br>Response format: 6 response options (doctor alone, myself alone, myself and the doctor together, doctor, myself and my family equally, doctor and my family together, myself and my family together)	Not reported
Kiessling [74]	[German translation and construct validation of the Patient-Provider-Orientation Scale (PPOS-D12)]	2014	Germany & Switzerland	Patient-Provider-Orientation Scale; German adapted version (PPOS-D12)	University/Medical schools	Medical students		396 (medical students)	To investigate the extent of patient-centredness of physicians and patients and to compare provider's and patient's congruence regarding patient centeredness	Based on:<br>(1) translation of english version by German-speaking physician and English-speaking ethnologist, subsequent consensus on item wording<br>(2) pilot testing with medical students<br>(3) check of construct validity using Cronbach's α and confirmatory factor analysis<br>(4) establishment of a new, shortened version of the German PPOS (PPOS-D12)<br>(5) analysis of construct validity with data from a second cohort of medical and dental students	Patient centredness:<br>sharing: attitudes towards sharing power, control, and information, patient involvement in decision-making<br>caring: practitioners engagement in psychosocial aspects of therapy and interest in their patients' expectations, wishes and life circumstances	Total: 12<br>Subscale 1: 6<br>Subscale 2: 6	2 subscales<br>1) Sharing (Partizipation)<br>2) Caring (Zuwendung & Interesse)<br>Prompt: statements<br>Response format: 6-point Likert-scale from 1 = 'I fully agree' to 6 = 'I don't agree at all'	Final version:<br>Total scale (patient centredness): Cronbach's α = .726<br>Subscale 1: Cronbach's α = .665<br>Subscale 2: Cronbach's α = .661<br><br>Other psychometric information:<br>Factor analysis
Krantz [75]	Assessment of Preferences for self-treatment and information in health care	1980	USA	Health Opinion Survey (HOS)	University	Students		349 (total)<br>Pilot 1: 200<br>Pilot 2: 159<br>(medical students)	To measure preferences for different treatment approaches	Based on:<br>(1) development of an item pool about aspects of medical care; partly adapted from another questionnaire<br>(2) pilot testing with undergraduates<br>(3) item analysis and reduction of item number<br>(4) pilot testing of remained items with another group of undergraduates<br>(4) analysis of construct validity using factor analysis to obtain components of the scale<br>(5) analysis fo discriminant validity by using other instruments<br>(6) analysis of test-retest analysis	Decision making preferences (active/informed versus inactive/trusting role)<br>Preferences for information and behavioral involvement in medical care	Total: 16<br>Subscale 1: 9<br>Subscale 2: 7	2 subscales:<br>1) Information (I): desire to ask questions and wanting to be informed about medical decisions<br>2) Behavioral Involvement (B): attitudes toward self-treatment and active behavioral involvement of patients in medical care<br>Prompts: statements<br>Response format: binary 'agree" - 'disagree'	Total HOS scale: KR 20: .77; test-retest reliability: .74<br>Subscale 1) I: KR 20: .74; test-retest r.: 0.59<br>Subscale 2) B: KR 20: .76; test-retest r.: .71<br><br>Other psychometric information:<br>discriminant validity
Kraetschmer [76]	Preferences of patients undergoing angiogram for participation in treatment decisions, coping style and the problem Solving Decision-Making scale (Doctoral dissertation)	1994	Canada	Problem Solving Decision-Making Scale (PSDM)	Hospital (Cardiovascular Investigative Unit)	Patients undergoing angiogram; nursing students	300	pilot 1: 10 (healthy people)<br>pilot 2: 69 (nursing students)	To examine patient preferences for involvement in treatment decision making and desire for information	Based on:<br>(1) Judgement of instrument in relation to scope and design by healthy people, physicians, academics and research methodologist (Content validity)<br>(2) factor analysis and reliability testing with nursing students	Theory of patient participation elements of choice behaviour:<br>problem solving (PS, identifying the one right answer)<br>decision making (DM, selecting the most desired bundle of outcornes)	Total: 18<br>3 vignettes with 2 subscales each<br>subscale 1) problem solving: 4<br>subscale 2) decision making: 2	Prompt: 3 vignettes:<br>Vignette 1: morbidity vignette<br>Vignette 2: mortality vignette<br>Vignette 3: quality of life vignette<br>with 6 items each<br>Subscale 1) problem solving tasks<br>Subscale 2) decision making tasks<br>Response format: single best with 5 response categories: 1 = 'doctor alone'; 2 = 'mostly doctor'; 3 = 'both equally'; 4 = 'mostly me'; 5 = 'me alone'	Vignettes: Cronbach's α ranging from .71 to .90 for 1 to 3 vignettes, respectively.<br>Subscale 1) problem solving tasks: test-retest reliability r = .46; paired t-test: (t=-0.61,df=20,p=0.514)<br>Suscale 2) decision making tasks: test-retest reliability r = .68; paired t-test: (t=0.31,df=21,p=0.76)
Krupat [24]	The practice orientations of physicians and patients: the effect of doctor-patient congruence on satisfaction	2000	USA	Patient–Practitioner Orientation Scale (PPOS)	Not-for-profit health services company	Primary care physicians and their patients	patient: 1020	Clinicians: 400	To investigate the extent of patient-centredness of physicians and patients and to compare provider's and patient's congruence regarding patient centeredness	Not reported in detail	Patient centredness with 2 dimensions:<br>sharing: attitudes towards sharing power, control, and information, and the extent to which patients should be involved in decision-making<br>caring: practitioners engagement in psychosocial aspects of therapy and interest in their patients' expectations, wishes and life circumstances	Total: 18<br>Subscale 1) sharing: 9<br>Subscale 2) caring: 9	2 subscales<br>1) sharing<br>2) caring<br>Prompt: statements<br>Response format: 6-point Likert-scale from 1 = 'I fully agree' to 6 = 'I don't agree at all'	Patient sample:<br>Total scale: Cronbach's α = .79<br>Subscale 1) sharing: Cronbach's α = .72<br>Subscale 2) caring: Cronbach's α = .52<br>Physician sample:<br>Total scale: Cronbach's α = .73<br>Subscale 1) sharing: Cronbach's α = .67<br>Subscale 2) caring: Cronbach's α = .52
Levinson [48]	Not all patients want to participate in decision making: a national study of public preferences	2005	USA		Households	English-speaking adults		2750 (general population)	To assess public preferences for participation	Based on:<br>(1) review of literatur and theoretical models and identification of items to measure different aspects of participation in decision making developed by other investigators.<br>(2) pilot testing of items previously developed<br>(3) Modification of items based on cognitive testing of a pilot questionnaire and feedback from respondents regarding clarity of wording and full understanding of the underlying concept	Information preferences (seeking and exchanging information, problem solving)<br>Decision making preferences	Total: 3	Prompt: statements<br>Knowlege: 'I prefer to rely on my doctor's knowledge and not try to find out about my condition on my own'<br>Options: 'I prefer that my doctor offers me choices and asks my opinion'<br>Decision: 'I prefer to leave decisions about my medical care up to my doctor'<br>Response format: 6-point-Likert scale ranging from 1 = 'strongly agree' to 6 = 'strongly disagree'	Not reported
Liebherz [77]	Information and decision-making needs among people with anxiety disorders: Results of an online survey	2015	Switzerland		Recruitment through online portal	Patients with anxiety disorders	60		To determine patients' information and decision-making needs as a pre-requisite for the development of patient decision aids for anxiety disorders	Based on:<br>(1) systematic literature and guideline search	Information preferences<br>Decision making preferences	Total: 23<br>Section 1: 3<br>Section 2: 2<br>Section 3: 2<br>Section 4: 16	4 sections:<br>1) Internet use<br>Prompt and response format not reported in detail<br>2) Online health information needs<br>Prompt: Question<br>Response format: 6 resp. 7 content related statements (yes/no)<br>3) Role in decision-making (items adapted from Control Preference Scale)<br>4) Treatment decisions (e.g. treatment setting, start of treatment, type of treatment)<br>Prompt: Question (have you ever made these decisions<br>Response format yes/no; if yes: assess the difficulty of these decisions on a 4-point scale from 'very difficult' to 'very simple'.<br>Additional free text fields.	Not reported
Lindig [78]	Adaptation and qualitative evaluation of Ask 3 Questions. A simple and generic intervention to foster patient empowerment	2020	Germany	Ask 3 Questions	Outpatient clinics of a comprehensive cancer center, inpatient setting	Patients with cancer	34 (total)<br>10 (phase 1: interviews)<br>24 (phase 2: focus groups)		To enhance SDM by patients' question-asking behaviour, physicians' provision of information about treatment options, their risks and benefits, and integration of preferences in decision-making	Based on translation and adaptation of English Ask3Q versions by following the team translation protocol TRAPD (Translation, Review, Adjudication, Pretesting and Documentation)<br>(1) translation into German by two members independently<br>(2) review of translation by a third bilingual team member,<br>(3) discussion of all translations and suggestions until reaching consensus on a final translation<br>(4) assessment of comprehensibility of all translated Ask3Q versions conducting cognitive interviews with patients+L27<br>(5) discussion of results and selection of a final version	Involvement in treatment decision making	Total: 3<br>(in addition to CPS)	3 questions on a postcard (german language)<br>1) Welche Möglichkeiten habe ich?<br>2) Was sind die Vor- und Nachteile jeder dieser Möglichkeiten?<br>3) Wie kann ich Unterstützung bekommen, um eine Entscheidung zu treffen?<br>Response format: open answers<br>(English translation by CK: 1) What options do I have? 2) What are the advantages and disadvantages of each of these options? 3) How can I get support to make a decision?)	Not reported
Luker [79]	The information needs of women newly diagnosed with breast cancer	1995	UK		University teaching hospital	Patients newly diagnosed with breast cancer	150		To establish the priority information needs of a sample of women newly diagnosed with breast cancer	Based on:<br>(1) literature search	Information preferences	Total: 9	Prompt: Women were asked to decide which of the two information needs had the greater importance. The procedure was repeated for all 36 pairs of information.<br>Information about 1) advance of disease; 2) likelihood of cure; 3) treatment affect on social activities; 4) disease affect family and close friends; 5) affect on myself at home; 6) treatment affect feelings about body and sexual attractiveness; 7) different types of treatment and advantages and disadvantages; 8) about risk for children or family members of getting breast cancer; 9) unpleasant side-effects of treatment	Not reported
Magnezi [80]	Would your patient prefer to be considered your friend? Patient preferences in physician relationships	2015	Israel		Housholds	General population		508 (general population)	To investigate relation between trust and shared decision making, and referred terminology	Based on:<br>(1) pilot testing with researchers<br>(2) reflection of researchers' comments using cognitive interviews<br>(3) pilot testing with 15 people (non-participants in the study) using telephone interview<br>(4) modifications based on results	Trust<br>Doctor–patient relationship<br>Preferred terminology as means of doctor-patient relationship	Total: 2	Prompt: 1 Vignette with 2 questions<br>Vignette: A family doctor has different ways of approaching a person who comes for medical service.<br>Question 1 (doctor–patient relationship): When you come to receive medical service from your family doctor, how do you feel s/he relates to you? Do you feel s/he approaches you mainly as...<br>Question 2 (How the interviewee would like to be approached by the physician): How would you like the doctor to relate to you?<br>Response format: single best with 6 options (patient, insured person, friend/peer of the doctor, medical service consumer, client that deserves medical services, partner in the medical treatment)	Not reported
Martinez [81]	Does physician communication style impact patient report of decision quality for breast cancer treatment?	2016	USA		Data from registries: Surveillance Epidemiology and End Results (SEER)	Patients with breast cancer	1690		To explore patient perceptions of autonomy-supportive communication from their surgeons and medical oncologists as well as patient-reported decision quality	Not reported in detail	Involvement in treatment decision making	Total 2	Prompt: statements ('When it came to getting treatment for my breast cancer, I preferred to be told what to do'; 'When it came to getting treatment for breast cancer, I wanted my doctor to tell me what to do')<br>Response format: 5-point scales from 'none of the time' to 'all of the time'	Not reported
Murtagh [82]	Evaluation and ethical review of a tool to explore patient preferences for information and involvement in decision making	2006	UK		3 hospices	Patients with advanced life-threatening illnesses and their doctors	110 (total)<br>61 (intervention group)<br>40 (control group)	no reported in detail (clinicians)	To explore patient preferences for information and involvement in decision making as a clinical tool	Based on:<br>(1) adapation of a questionnaire published elsewhere	Information preferences about illness and treatment<br>Preference for family/carer information;<br>Decision-making preferences<br>(Satisfaction with amount of information, the way information had been given, with information given to the family or carer<br>Confidence about future decision making, matching their preferences<br>Previous experience of information about the illness)	Total 8	Prompt: questions<br>Response format: different types; yes/no, 3-point-scales, 6-point-sclae, free text	Not reported

Neumann [83]	Identifying and predicting sub-groups of information needs among cancer patients: An initial study using latent class analysis	2011	Germany	Cancer Patients Information Needs (CaPIN)	University hospital	Cancer patients	323		To increase awareness of oncology nurses and physicians of the complexity and heterogeneity of information needs among Cancer Patients	Based on:<br>(1) in-depth interviews with patients	Information preferences	Total: 23<br>Subscale 1: 4<br>Subscale 2: 3<br>Subscale 3: 7<br>Subscale 4: 8<br>1 open question for free text	4 dimensions:<br>1) Information from the physician: medical examination results and treatment options<br>2) Information from the physician: side effects and medication<br>3) Information regarding social issues<br>4) Information regarding health promotion<br>Prompt: Questions (Looking back on your hospital stay, would you have liked more information about...)<br>Response format: binary: 0 = no, 'I would have liked no more information', 1 = yes, 'I would have liked more information'	Total CaPIN: Cronbach's α = .90<br>Subscale 1: Cronbach's α = .82<br>Subscale 2: Cronbach's α = .78<br>Subscale 3: Cronbach's α = .78<br>Subscale 4: Cronbach's α = .85
Pauli [84]	A short scale for measuring attitudes towards the doctor-patient relationship: psychometric properties and measurement invariance of the German Patient-Practitioner-Orientation Scale (PPOS-D6)	2021	Germany	Patient-Practitioner-Orientation-Scale; German adapted version (PPOS-D6)	University	Medical & dental students		332 (medical students)	To investigate the extent of patient-centredness of physicians and patients and to compare provider's and patient's congruence regarding patient centeredness	Based on:<br>(1) adaptation of a questionnaire published elswhere by eliminating semantically redundant items or items less related to the underlying concept in order to develop a short version of the scale	Patient centredness with two dimensions:<br>Sharing: attitudes towards sharing power, control, and information, and the extent to which patients should be involved in decision-making<br>Caring: practitioners engagement in psychosocial aspects of therapy and interest in their patients' expectations, wishes and life circumstances	Total: 6	Prompt: statements<br>Response format: 6-point Likert-scale from 1 = 'I fully agree' to 6 = 'I don't agree at all'	For total scale and both subscales only narrative decription: "Cronbach's α indicated poor internal consistency"
Puschner [85]	Development and psychometric properties of a five-language multiperspective instrument to assess clinical decision making style in the treatment of people with severe mental illness (CDMS)	2013	Germany, UK, Italy, Denmark, Hungary, and Switzerland	Clinical Decision Making Style Scale(CDMS)	Outpatient/community mental health services at six centres throughout Europe	Patients with severe mental illness; mental health professionals	588	213 (mental health professionals)	To explore patients' preferences for two dimensions of autonomy: decision making and information seeking for use in mental health care	Based on:<br>published principles for the translation and cultural adaptation of PROMs identifying 10 sequential steps: 1 preparation; 2 forward translation; 3 reconciliation; 4 back translation; 5 back translation review; 6 harmonisation; 7 cognitive debriefing; 8 review of cognitive debriefing results and finalisation; 9 proof-reading; and 10 final report<br>Focus grous in step 1 & 7	Information preferences<br>Decision making preferences	Total: 21<br>Section A: 6<br>Section B: 9<br>Section C: 6	3 sections:<br>Section A: general preferences regarding patient autonomy in decisions<br>Section B: decision making preferences in 3 scenarios (3 items per vignette)<br>Section C: desire for information<br>Prompt: section A & C: ; section B: vignettes<br>Response format: section A and C: 5-point Likert scale from 0 = 'strongly disagree' to 4 = 'strongly agree'; section B: CDMS-P scored from 4 = 'Me' 0 = 'Clinician') in , section B CDMS-S scored from 4 = 'Service user' to 0 = 'Me'	Patient version<br>CDMS-P PD: Cronbach's α between .87 and .89<br>CDMS-P IN: not reported<br>Staff version<br>CDMS-P PD: Cronbach's α ranged between .87 and .89<br>CDMS-P IN: not reported
Ratcliffe [86]	Assessing patients preferences for characteristics associated with homeopathic and conventional treatment of asthma: a conjoint analysis study	2002	UK		Outpatient clinic of two hospitals	Adult patients with asthma	300		To investigate the preferences of patients with asthma for attributes or characteristics associated with treatment for their asthma	Based on:<br>(1) semi-structured interviews with patients<br>(2) formulation of attributes based on interview results<br>(3) development of levels for each attribute by the research team to reflect plausible ranges reflecting realistic levels for patients receiving treatment for their asthma	Extent to which the doctor gives sufficient time to patients concerns and discussing treatment options;<br>Extent to which the doctor treats the patient as a whole person	Total: 17<br>Section A: 9 (relevant to patient preferences)<br>Section B: 8 pairwise choices	3 main sections<br>1) section A<br>Prompt: respondents were asked to indicate the degree of importance of nine characteristics of services for asthma treatment<br>Response options: 3-point scale from 'very important' to 'not very important'<br>2) section B: 8 pairwise choices<br>3) section C: sociodemographic details	Not reported
Spies [87]	Preferences for shared decision making in chronic pain patients compared with patients during a premedication visit	2006	Germany	Autonomy Preference Index; German version (API)	University hopistal (Premedication visit, chronic pain clinic)	Patients with chronic pain	341		To explore patients' preferences for two dimensions of autonomy: decision making and information seeking.	Based on<br>(1) translation of API (no further information)	Information preferences<br>Decision making preferences	Total: 14<br>Subscale SDM: 6<br>Subscale IS: 8	2 subscales<br>1) decision making scale SDM<br>2) information seeking scale IS<br>Prompt: statements<br>Response format: 5-point Likert scale. Response choices range from 'strongly disagree' to 'strongly agree'	Subscale 1 SDM<br>Cronbach's α = 0.61 (chronic pain clinic)<br>Cronbach's α = 0.64 (premedication visit)<br>Subscale 2 IS<br>Cronbachs' α = 0.73 (chronic pain clinic)<br>Cronbach's α = 0.66 (premedication visit)
Strull [88]	Do patients want to participate in medical decision making?	1984	USA		Outpatient clinic of a community hospital; free-standing health maintenance organization (HMO), outpatient clinic	Patients with hypertension	210	50 (clinicians)	To explore the amount of information patients prefer about their disease and its therapy, and preferred involvement in treatment decisions	Not reported in detail	Information preferences about hypertension and its therapy preferences of involvement in treatment decisions	Total: 4	Prompt: Some people want clinicians to make all decisions about medicines based only on the best medical practice. Other people also want the clinician to ask them their opinion about the decision. Finally, some people want to make the decision themselves, after getting the clinician's advice or opinion.'<br>4 categories with 1 item each:<br>1) Information about hypertension<br>Response format: 5-point-scale (e.g. all there is to know)<br>2) Preferences for roles in Decision making<br>Response format: 5-point-scale (e.g. Clinician should make the decision but strongly consider patients opinion)<br>3) Amount of discussion about therapy<br>Response format: 4-point-scale from 'none' to 'very extensive'<br>4) How decisions are usually made<br>Response format: 4-point scale (e.g. Clinician makes the decision, using all that's known about the medicines)	Not reported
Sutherland [89]	Cancer patients: their desire for information and participation in treatment decisions	1989	Canada	Health Opinion Survey (HOS), Information Seeking Questionnaire (ISQ), Preference for participation in treatment decisions questionnaire	Ambulatory care facility	Patients with cancer	52		To explore patients preferences for information needs and involvement in treatment decisions	Based on:<br>(1) selection of items of a questionnaire published elswhere<br>(2) discussion with patient panel to verify and/or suggest additional items	Information preferences<br>Preference for involvement in treatment decisions	HOS<br>Total: 16<br>B Scale: 9<br>I scale:7<br>ISQ<br>Total: 18<br>Preference for participation in treatment decisions questionnaire<br>Total: 5	3 scales<br>Health opinion Scale (HOS)<br>2 subscales: behavioral involvement = B scale; information= I scale<br>prompt: statements<br>Response format: A binary agree/disagree format Information Seeking Questionnaire (ISQ)<br>Linear analog self-assessment (LASA): different types of information that a patient would like to have about cancer and its treatment, more global nature (overall assessment of amount, detail, andunderstanding of information,degree of active information-seeking, satisfaction with details acquired)<br>Preference for participation in treatment decisions questionnaire:<br>adapted from a questionnaire published elsewhere<br>Prompt: patient's preference for participation in treatment decision making under 'ideal' circumstances; and patient indicated how his/her treatment decisions were 'actually' made. Response format: 5-point rating scale from the physician assuming full responsibility for decision making to the patient assuming this role	Health Opinion Survey (HOS):<br>Total scale: KR 20 = .74<br>I subscale: KR 20 = .68<br>B subscale: KR 20 = .68<br>ISQ: KR 20 = .88<br>Preference for participation in treatment decisions questionnaire: not reported
Xie [90]	Health information and decision-making preferences in the Internet a age: a pilot study using the Health Information Wants (HIW) Questionnaire	2010	USA	The Health Information Wants Questionnaire (HIW)	State university (public library computer class)	Undergraduate students in a variety of disciplines;<br>older adults		43 (total)<br>22 (students)<br>21 (adults)	To measure preferences for health information and participation in health decision-making to improve patient-provider relationships and the quality of health care.	Based on:<br>(1& 2) development of items based on literature search and an exploratory study<br>(3) content validity testing<br>(4) cognitive testing<br>(5) pilot study testing<br>authors focus on s on Stage 5	Information Preferences<br>Decision-making Preference	Total: 40 topic with 2 questions each = 80 items<br>Subscale 1: 40<br>subscale 2: 40	2 subscales with 7 sub-scales with parallel items to the following topics:<br>diagnosis (6 items); treatment (9 items); laboratory test (4 items); self-care (5 items); complementary and alternative medicine (CAM) (6 items); psychosocial (8 items); health care provider (2 items);<br>Subscale 1) Information Preference Scale<br>Prompt: indicate preferences for each type of information<br>Response format: 5-point Likert scale from 1 = 'none' to 5 = 'all'<br>Subscale 2) Decision-making Preference Scale<br>Prompt: indicate preferences for each type of health decision-making<br>Response format: 5-point Likert scale from1= 'the doctor alone' thorugh 'mostly the doctor', 'the doctor and myself equally', 'mostly myself', to 5 = 'myself alone'	Information Preference Scale:<br>Total scale: Cronbach's α = 0.98<br>Subscales ranging from Cronbach's α = .89 to .98 (mean .95)<br>Decision-making scale:<br>Cronbach's α = 0.96<br>Subscales ranging from Cronbach's α = .75 to .96 (mean .89)
